# Evaluation of bone regeneration in mandible large defect using undifferentiated adipose stem cells loaded on gelatin carrier: An animal model case study

**DOI:** 10.34172/joddd.2021.005

**Published:** 2021-02-13

**Authors:** Ali Hossein Mesgarzadeh, Islam Nasiri, Seyedhosein Jarolmasjed, Mehran Naghibi, Hajar Shafaei

**Affiliations:** ^1^Department of Oral and Maxillofacial Surgery, Faculty of Dentistry, Tabriz University of Medical Sciences, Tabriz, Iran; ^2^Department of Anatomical Sciences, Faculty of Medicine, Tabriz University of Medical Sciences, Tabriz, Iran

**Keywords:** Bone regeneration, Stem cell, Defect

## Abstract

**Background.** Large mandibular defects are considered difficult reconstructive challenges for oral and maxillofacial surgeons. Cell therapy, as an alternative technique, might increase the speed of bone regeneration. This study aimed to investigate bone regeneration in large defects of dog mandibles using allogenic adipose-derived stem cells on gelatin foam as a cell carrier.

**Methods.** The tissue engineering phase consisted of the sampling of adult dogs’ adipose tissue that can easily be isolated from adipose stem cells (ASCs) of the dogs, ASCs were cultured in Dulbecco’s Modified Eagle’s Medium (DMEM, Gibco, USA) with low glucose, containing 10% fetal bovine serum (FBS) (Sigma, USA) and 1% penicillin-streptomycin (Gibco, USA), with the characterization of dog ASCs and gelatin-transplanted ASCs. Six dogs were included in this experimental study in the next step and randomly assigned to the treatment and control groups. The samples in both groups underwent surgery under general anesthesia to create uniform 3-cm bony defects. The samples in both groups were reconstructed with titanium reconstruction plates and screws. A large bone gap filled with ASCs (5×10^6^ ) was seeded on gelatin (ASCs) in the treatment group. In the control group, bony defects were filled with a cell delivery carrier without ASCs. Six months after transplantation, the animals’ mandibles were evaluated by CT scan imaging, and the results were quantified through the Hounsfield unit (HU). The data were analyzed with t-test.

**Results.** Before transplantation, the nature of the stem cells was confirmed by the expression of CD44 and CD105 cell markers at 71.9% and 89.3%, respectively, and a lack of the CD45 cell marker expression at 2.2%. Evaluation of CT scan images showed significantly higher bone repair in the ASCs group (920.25±572.92 HU) than in the control group (-94.746± 08.42).

**Conclusion.** The bone regeneration of the ASCs group was significantly higher than that in the control group.

## Introduction

Large bone defects are a major clinical challenge due to delayed or incomplete bone healing. Small bone injuries can be reconstructed spontaneously. However, if the bone defects go beyond the critical size defect, they will not heal without therapeutic intervention. These critical size defects might be induced by trauma, infection, and tumor resection, and require bone grafts for reconstruction.^1^

As much as 5%–10% of bone fractures cannot be repaired and reconstructed into the previous normal shape. Currently available bone grafts at the clinics include autografts, allografts, xenografts, and synthetic biomaterials. Autograft is the gold standard and has osteogenic, osteoconductive, and osteoinductive features. Despite the satisfactory results of bone reconstruction with autografts, these types of grafts are associated with complications and transmission of infection at the graft resection site and donor site morbidity, limiting its application.^2^

The disadvantages of allografts include the risk of immunological rejection, transmission of infectious diseases, and adverse effects on the graft’s mechanical and biological properties.^3^ Numerous studies have been conducted on alternative natural or synthetic materials to overcome bone grafts’ common therapeutic limitations.^4,5^ Alternative bone grafts should be biocompatible and osteoinductive, structurally resemble natural bone, be easy to use, and be available. They must also pass safety and efficacy tests both in vivo and in vitro.^6^

For tissue engineering purposes, Johnston differentiated bone marrow mesenchymal stem cells toward chondrogenesis for the first time, and others used it for osteogenesis as well.^7,8^ Recently, other sources of mesenchymal cells, such as muscle, skin, adipose tissue, synovial membrane, dermis, trabecular bone, periosteum, pericytes, and blood have also been identified.^9^

Recently, adipose stem cells (ASCs) have become popular mesenchymal stem cells. There are differences and similarities between ASCs and bone marrow-derived stem cells (BMSCs).^10,11^ The isolation and differentiation of ASCs were performed by Zuk et al.^12,13^ The abundance of adipose tissues in the body and the ease of sampling are the main advantages of ASCs. ASCs also proliferate at a higher rate in culture medium than BMSCs.^9^ Nevertheless, one of the main barriers to harvesting MSCs from bone marrow might be infection or septicemia complications.^14^ On the other hand, these two types of cell sources are also different in the number of stem cell yield, where the number of stem cells in 100 g of adipose tissue is 300 times higher compared to 100 mL of bone marrow.^15,16^

The effectiveness of mesenchymal stem cells has been shown in canine animal models for increasing bone formation.^17^ Sheikhi et al^18^ did not observe any statistically significant differences between two autogenous bone graft and tissue engineering methods by ASCs compared to bone density repaired in the alveolar cleft of the dog maxilla. A study used pre-formed plates and autologous graft of the iliac bone for reconstructing a 40-mm defect of the mandibular body of nine hybrid dogs.^19^ Recently, a study reported that ASCs are more osteogenic than dental pulp stem cells for the regeneration of mandibular defects in rats.^20^ Bone tissue engineering, using BMSC in hydroxyapatite and tricalcium phosphate, exhibited an osteogenic potential.^21^ However, these materials have induced osteogenesis.^22^

Many studies have used osteogenesis inducers, such as bone morphogenic protein-2 (BMP-2), to differentiate MSCs.^23,24^ For the clinical application of BMP-2, as a potent osteoinductive cytokine, more investigations are required to determine its safety.^25^ It was shown that undifferentiated or differentiated BMSC transplantation had no significant differences in the treatment of liver cirrhosis.^26^ It is believed that BMSCs produce some cytokines,^27^ and BMP-2 promotes bone regeneration28 in conditioned media. Inadequate celltherapy studies are available on the regenerative potency of undifferentiated ASCs to treat bone defects. Thus, this study aimed to evaluate bone regeneration of large defects in dog mandibles using undifferentiated ASCs.^29,30^

## Methods

This experimental study was carried out at the Tissue Engineering Laboratory of Medical School, Tabriz University of Medical Sciences. All the stages of this study conformed to the guidelines of the Organizational Ethics Committee of Tabriz University of Medical Sciences for animal studies. Furthermore, laboratory safety principles were observed to prevent contamination in the laboratory environment or any damage to colleagues. The canines were kept under standard conditions with easy access to water and food.

### Sampling and isolation of ASCs

Tissue samples were provided from fresh, visceral adipose tissue of a male dog. The donor male dog was anesthetized with ketamine (5.5 mg/kg) and diazepam (0.3 mg/kg), and the omental adipose tissue was removed. The obtained adipose tissue sample was washed with phosphate-buffered saline solution (PBS) containing 1% penicillin-streptomycin and chopped into 2–3-mm pieces with a scalpel blade and then washed several times with PBS to remove excess tissues, such as blood cells and connective tissue. After measuring the samples’ net weights, based on the weight, collagenase-1 degrading enzymes were used to digest the tissues. Collagenase 1 enzyme (0.5 mg) (Gibco, USA) was added to the container with samples per gram of the tissue for one hour. Isolated cells were seeded into each flask (T25) with 5 mL of culture medium added to each flask and finally incubated with CO2 at 37°C. The cell culture medium was changed every 72 hours. Once the cells reached 70% confluence, they were passaged to perform the re-culturing process up to three passages.

### Flow cytometry immunophenotyping

ASCs (10^5^ cells) of the third passage were prepared in specific flow cytometry tubes for each CD marker. The cells were washed in a flow cytometry tube with PBS containing 1% BSA. In the next step, each tube was labeled with 3 µL of CD105 (cat: 562759 BD Biosciences, San Jose, CA, United States) with phycoerythrin fluorescent material and FITC-labeled CD44 antibody (cat: 560977, BD Biosciences, San Jose, CA, United States), CD45 (cat: 560976, BD Biosciences, San Jose, CA, United States) with a fluorescent material (FITC). The cells were kept within the tubes for 30 minutes in a dark environment at room temperature. After the incubation time, the cells were washed twice with PBS containing 1% BSA. The cells were gently vortexed with 0.5 mL of PBS containing 1% BSA and analyzed by flow cytometry. One of the tubes was kept as an unstained cell sample. WinMDI software 2.8 was used for data analysis.

### Preparation of cells for transplantation

**Figure 1:**
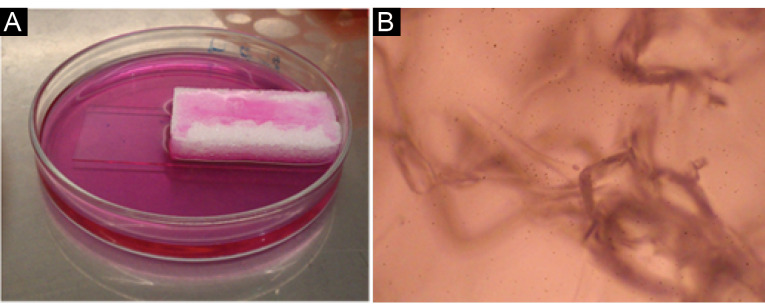
ASCs on gel foam. A) Wetting gel foam with working DMEM. B) Microscopic view of wetted gel foam under inverted microscope (×40).

Gel foam was placed in the working medium to remain wet for 15 minutes. ASCs (5×10^6^) were seeded on commercial surgical gel foam (Gelita-Spon, Gelita Medical, Germany). Then, the cells were added on prewetted gel foam (Figure 1A). ASCs cell suspension was gently placed on gel foam, and the cells gradually penetrated the gel’s pores. This construct was transported to the animal operation room under a sterile condition and placed between the two unwetted gel foams (sandwich model) for easy placement in the defect area. ASCs were loaded on surgical gel foam, on the day of surgery.^13,31^

### Surgery

Surgery was performed on six 1–2-year-old male native dogs. The animals weighed 20–25 kg. Before surgery, the dogs received antiparasitic treatment. Blood biochemistry, hematologic tests, and calcium and phosphorus serum levels of the animals were normal. The surgical area’s skin was shaved and prepared; under general anesthesia, a 10-cm cutaneous incision was made on the left mandible. After dissection, the periosteum was elevated and the mandibular bone was exposed. Then, an intraoral mucoperiosteal envelope flap was created using a gingival sulcular incision from the distal area of the canine tooth to the mesial gingival sulcus of the first molar. The bone was resected up to 3-cm length from the alveolar crest to the mandibular lower border from the second premolar (P2) to the first molar (M1) with tungsten carbide surgical burs measuring 1.2 mm in diameter under normal saline solution irrigation.

Temporary intermaxillary fixation was carried out, and the gap was reconstructed with an 8-hole titanium reconstruction plate (Imen Ijaz, Iran) with five screws measuring 2.7 mm in diameter (Imen Ijaz, Iran).

**Figure 2:**
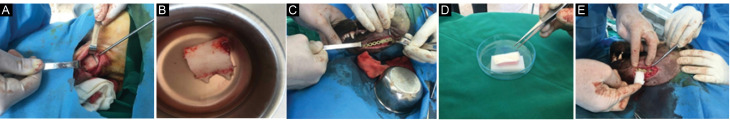
Surgery steps. (A) Extraoral access. (B) Resected bone fragment. (C) Placing the plate. (D) Transplanted ASCs on the gel foam. (E) Placing ASCs on the gel foam in defect.

The bony gap was filled with gel foam alone and ASCs-loaded gel foam, respectively, in the control and treatment groups. Intraoral incisions were sutured with vicryl sutures using a water-tight technique. Extraoral incisions were sutured in tissue layers with resorbable and nylon sutures (Figure 2).

ASCs were loaded on gel foam on the day of surgery, filling the defect, as illustrated in the video file. The animals received cefazolin (25 mg/kg/12 h) for a week, amikacin (15 mg/kg/24 h) for five days, and ketorolac (0.5 mg/kg/24 h) for three days postoperatively.^32^

### Determining ossification

Six months after the surgery, all the groups underwent CT scan examinations (Siemens, Germany). The dogs were sedated in a supine position on the CT scan bed, and each animal’s head was fixed in the standard position on the head holder. Then, the CT scan was performed with 6-mm sagittal and axial cuts in the animal jaw area. The images from the treatment and control groups were selected from the same sections, and the Hounsfield units (HUs) were calculated in eight areas for each image. HUs are standard numbers originating from CT imaging. HU represents the relative density of body tissues according to a calibrated gray-level scale, based on values for air (-1000 HU), water (0 HU), and bone density (+1000 HU).^2^ Many studies have evaluated the use of HU to assess the relative bone density of the jaws in CT, and HU seems to be a useful method to analyze bone density.^33^

### Statistical analysis

Statistical analysis was performed by GraphPad Prism software. Independent *t* test was used for comparisons at *P*< 0.05 as a level of statistical significance.

## Results

### Spindle-shaped morphology of ASCs

**Figure 3:**
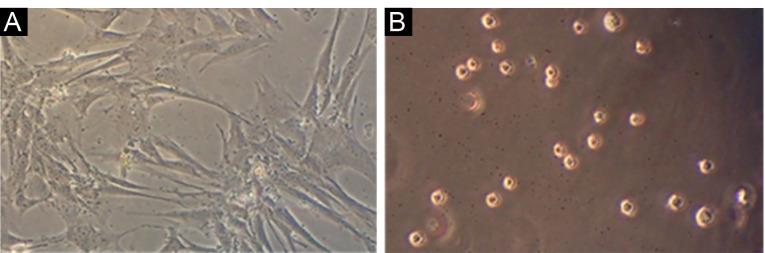
Photomicrograph of ASCs. A) Spindle-shaped ASCs at the third passage under an inverted microscope before transplantation. B) Homogenous round shape ASCs on hemocytometer slide under inverted microscope (Magnification ×100).

Images provided by an inverted microscope revealed that the cells had spindle-shaped morphology in the primary culture (Figure 3A). The spindle-shaped cells had a faster growth rate than other cells and formed the highest cell percentage. Before cell transplantation, Figure 3B shows the homogeneous population of round-shaped cells with specified nuclei, which is the typical morphology of mesenchymal stem cells at the third passage.

### Flow cytometry results

**Figure 4:**
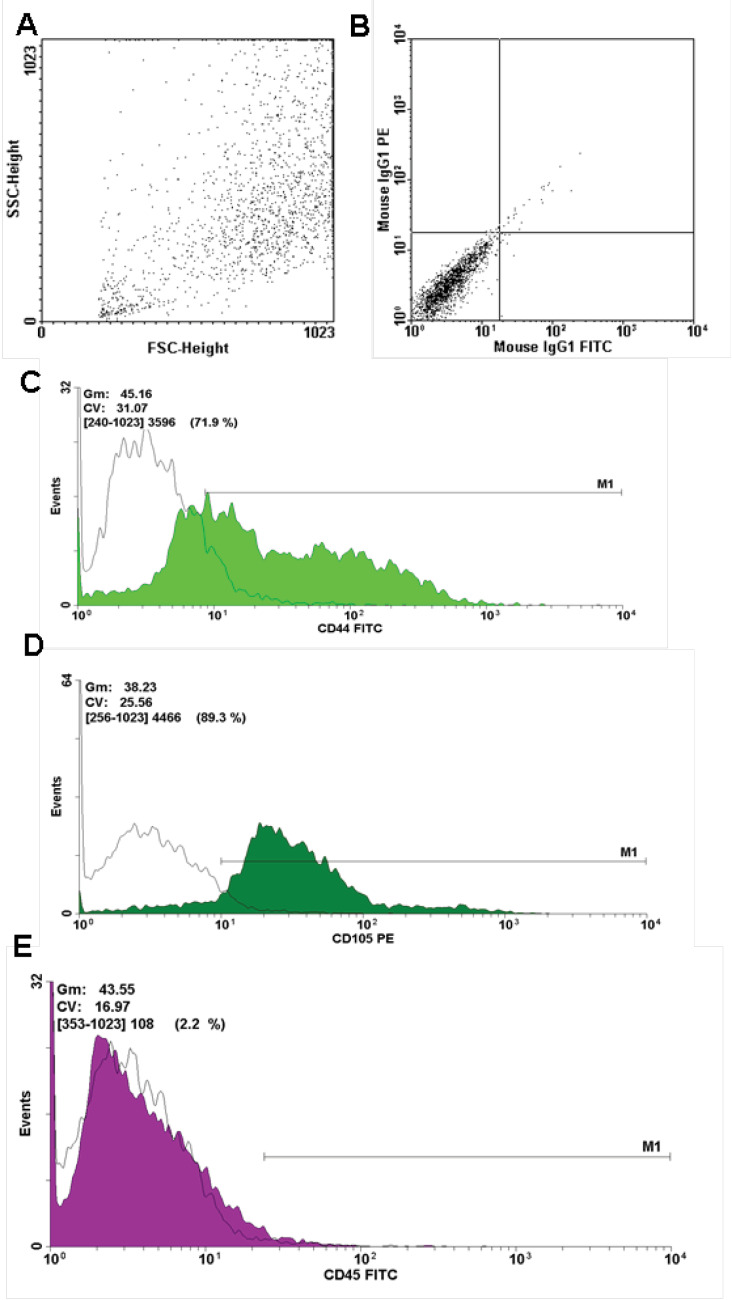
Flow cytometry results of adipose stem cells. A) Forward scatter dot plot shows homogenius transplanted cell. B) Isotype control is not expressed. C) The expression of positive CD44 CD surface marker. D) The expression of positive CD105 surface marker. E) The low expression of negetive markers..

The forward scatter and side scatter of ASCs in the dot blot diagram showed homogenous size and granularity of cells in the third passage (Figure 4 A). The dot blot diagram of Figure 4B illustrates the isotype control of IgG for normalizing specific CD markers. The expression rates of CD44- and CD105-specific mesenchymal markers were 71.9% and 89.3%, respectively (Figure 4C, D). The expression rate of hematopoietic marker CD45 was 2.2% (Figure 4E).

### Evaluation of bone regeneration by CT scan

**Figure 5:**
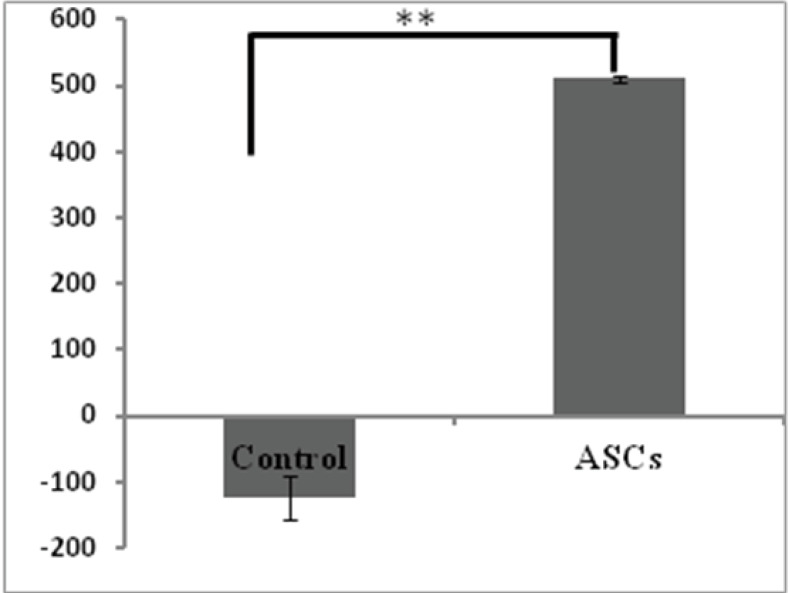
Comparison of the bone density of Hounsfield unit in two groups (control and ASCs). Sample size for control=3, sample size for ASC=2 (one of animals was not included in study). Data are presented Mean ± SEM (*P*< 0.001).

**Figure 6:**
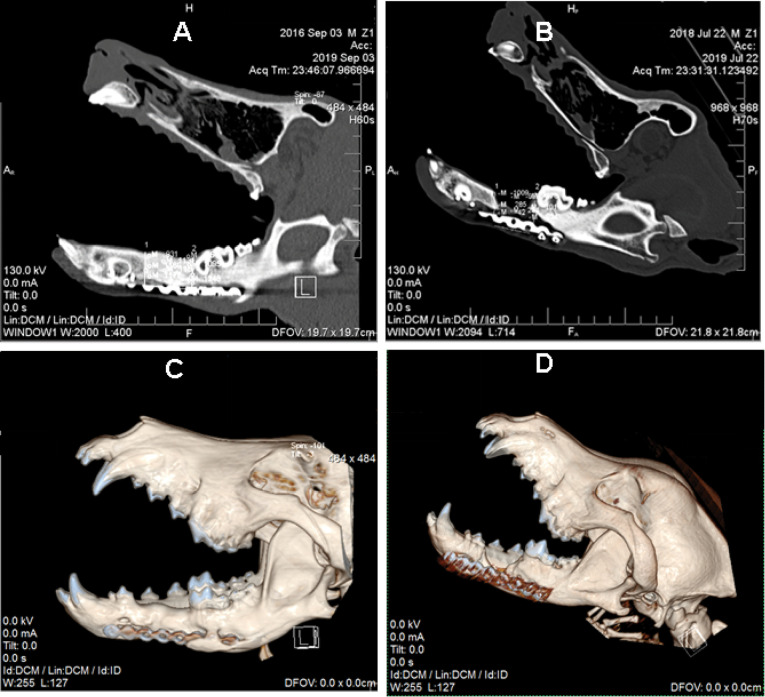
CT scan images of canine mandible. (A) Sagittal section of the ASCs group. (B) Sagittal section of control group. (C) 3D reconstruction of canine skull of the ASCs group. (D) 3D reconstruction of canine skull of the control group.

After six months, the samples were examined through a CT scan at the defect site for bone regeneration rate evaluation. One dog in the ASCs group was excluded from the study because of infection. The comparison of bone density with HU showed that it was -123.6 ± 32.1 HU in the control group and 511 ± 5.1 HU in the ASCs group (Figure 5). According to the t-test, a significant difference (*P*< 0.001) was observed in the bone density of these two groups. Figure 6 illustrates CT scan images of mandibular canines in the treatment and control groups. Bone formation is evident in the treated group compared to the control group (Figure 6A, B). In the 3D construction of the CT image, the ASCs group exhibited a significantly higher bone density than the control group (Figure 6C, D).

## Discussion

In bone regeneration, MSCs are promising candidates for tissue engineering. In the present study, homogeneous ASCs with defined characteristics of MSCs were transplanted. Transplantation was carried out with simple FDA-approved foam gel as this spongy gel is routinely used in surgeries as a blood absorbent and a coagulation agent in the surgical field. It is suggested for future cell therapy clinical trials as a cell carrier. We studied the effect of ASCs on bone regeneration without osteoinductive materials. We also observed promising results in accelerated bone regeneration of large defects of mandibular canines by ASCs in less than 6 months.

In the present study, cultured ASCs were obtained from visceral adipose tissue. It has been shown that the osteogenic potential of visceral ASCs was higher than subcutaneous ASCs^34^ and lower than the adipogenic potential of visceral ASCs.^35^ In this study, the ASCs transplanted expressed cell surface markers similar to other studies.^36^ We found that visceral ASCs are efficient in bone repair. In line with our observation, other studies have found no significant differences between visceral ASCs and subcutaneous ASCs in surface CD marker expression and cell viability.^37^ In this study, thin dogs were selected for adipose tissue sampling due to the impaired potency of cell proliferation of subcutaneous and visceral ASCs derived from obese than non-obese donors.^38^ Also, the findings showed that cultured ASCs had appropriate morphology and proliferation described for ASCs. According to our previous study, the spindle-shaped appearance of ASCs indicates the non-stem cell property of mesenchymal stem cells.^39^ Morphologically, ASCs in the culture are seen as spindle-shaped cells and flat cell subpopulations.^35^ Spindle-shaped MSCs have been introduced as useful for clinical purposes due to non-stem cell properties.^40^ Thus, homogeneity of ASCs in the current study probably provided favorable results from these cell therapy studies. Using homogeneous cells is one of the standards of ASCs therapies.^41^

On the other hand, the osteogenic potential of ASCs in the present study might correlate with the visceral origin of adipose tissue. The therapeutic mechanism of ASCs in the transplanted area can be related to these cells’ immunomodulatory effect.^29^ It has been shown that bone healing initiates after injury by proinflammatory cytokines. However, continuous expression of these cytokines can adversely affect healing.^42^ It seems that the presence of ASCs in the defect site in the present study might modulate bone regeneration by anti-inflammatory properties.

One of the challenges in clinical applications of MSCs is the allogeneic origins of cells; nevertheless, the present study was performed on allogeneic ASCs, and there was no transplant rejection. This might be attributed to the low immunogenicity of allogeneic MSCs.^43^ However, the biosafety of MSCs is discussed in regenerative medicine.

In the present study, after 6 months, all the groups were examined by CT scan for investigating bone regeneration rate in the defect site, where the bone density in the control group was significantly lower than that in the ASCs group.

Cui et al^44^ demonstrated that ASCs-seeded coral scaffolds were effective in repairing the 20×20-mm canine cranial bone defects. In a study by Abukawa et al,^45^ autologous porcine stem cells were seeded in D,L-lactic-co-glycolic acid and transferred to mandibular defects. They reported that the repaired bone in the defects treated with stem cell-seeded scaffolds was indistinct from native bone radiographically, but the control group’s defects remained radiolucent. Histologic assessment of regenerated bone in the mandibular defect of canines showed that bone-like tissue formed in animals treated with ASCs, but in the control group with the unseeded scaffold, ossification was limited to the periphery of the defect.

Haghighat et al^46^ isolated ASCs from subcutaneous fat of the four dogs’ lateral thoracic area and transferred them into cylinders measuring 9 mm in diameter through the mandibular defect. After six weeks, biopsies were taken, and histomorphometric analysis of the percentage of new bone formation was performed in each case, revealing no significant difference between ASCs-seeded and cell-free scaffold groups in terms of the percentage of bone formation. This minor and insignificant difference might be due to insufficient healing time before the biopsy procedures. The current study used visceral ASCs on cell delivery carriers instead of a scaffold.

In another study, the resection site of mandibular odontogenic cysts was filled with ASCs whose osteogenic properties had been induced by BMP-2, and results similar to this study were achieved. However, ASCs of our study had no osteogenic differentiation because a limited differentiation growth factor for clinical applications is preferred. In this study, the use of undifferentiated ASCs without any growth factor had some advantages: (i) minor in vitro manipulation of cells with potent growth factor, such as BMP2; (ii) using promising one-step procedures during surgery and transplantation of isolated ASCs; and (iii) probably osteogenic signals from the microenvironment of injured area or transplanted ASCs. Proteomic techniques revealed that MSCs produce a large number of cytokines to support tissue regeneration.^47^ Application of ASCs in bone regeneration recruited osteoblasts and osteoclasts of the host by producing signaling factors, such as vascular endothelial growth factor (VEGF), to promote angiogenesis.^48^

Regarding bone density, the present study revealed that bone regeneration in the large bony defect site was denser than the adjacent normal bone after six months. Yamada et al^49^ observed the efficiency of canine bone marrow stem cells, canine dental pulp stem cells, and puppy deciduous tooth stem cells with PRP scaffold in treating lesions on both sides of mandibular canines. The results of the above studies are consistent with the present study. In the present study, the ASCs group exhibited the highest bone density. Note that in the present study, there was no regeneration in the control group lesions.

Bohnenblust et al^50^ reported that the use of ASCs does not affect the repair of rat skull bone lesions. Bone density after repair in the allograft group alone and allograft with stem cells was similar at 1365±160.4. Despite Bohnenblust et al’s findings, the bone was denser in the defect in the present study than in the adjacent normal bone.

Different methods have been employed for measuring bone regeneration, including bone hardness rate, the percentage of regenerated bone, biomechanical evaluation, and CT scan methods. Iino et al^51^ compared bone repair after grafting in the CT scan and intraoral radiography. They claimed that the CT scan offered a better evaluation than intraoral radiography. While they compared bone height, the present study evaluated bone density. Due to the radiographic shortage in bone regeneration evaluation, a CT scan was used in this study.

Ihan Hern and Miljavec^52^ evaluated the spontaneous improvement of bone using the bone density method in digital radiography. They concluded that the final bone density increased over time, more clearly in small defects than in larger ones. According to our study, after 6 months, the regenerated bone density was higher than the normal bone in large defects.

Reportedly, the loading cells promoted mandibular regeneration using osteoblasts on beta-tricalcium phosphate versus beta-tricalcium phosphate in a canine model.^53^ Differentiation of BMSCs seeded into beta-tricalcium phosphate scaffold resulted in osteogenesis in mandibular canines.^54,55^ It has been shown that BMSCs labeled with a green fluorescent protein could be involved in bone repair.^56^

Further investigations are required to compare classic bone scaffolds with simple cell carriers in the present study and compare differentiated and undifferentiated ASCs for bone regeneration, though differentiated and undifferentiated ASCs equally promote nerve regeneration.^45,57^

## Conclusion

The present study showed significant bone formation in extra-large bony defects using ASCs loaded on commercial surgical gel foam as a cell carrier. It was concluded that tissue engineering might be an alternative technique to obtain favorable ossification in large bony defects in the maxillofacial region.

## Authors’ Contributions

AHM, HS, and SHJ contributed to the design of the study. HS performed in vitro experiments. AHM, IN, and SHJ performed the surgeries. MN contributed to CT scan imaging. AHM and HS performed data analysis and interpretation of data. IN wrote the manuscript.

## Acknowledgments

The authors thank the Medical Microbiology and Nanotechnology Department of Tabriz University of Medical Sciences.

## Competing Interests

No conflict of interest in terms of scientific collaboration and financial benefits is declared by the authors of the study.

## Ethics Approval

The Ethics Committee of Tabriz University of Medical Sciences approved the study protocol, with the ethics code of IR.TBZMED.VCR.REC.1398.260.
